# Composite Structural Supercapacitors: High-Performance Carbon Nanotube Supercapacitors through Ionic Liquid Localisation

**DOI:** 10.3390/nano12152558

**Published:** 2022-07-25

**Authors:** Benjamin Mapleback, Vu Dao, Lachlan Webb, Andrew Rider

**Affiliations:** Defence Science and Technology Group, Platforms Division, 506 Lorimer St, Melbourne, VIC 3207, Australia; vu.dao@defence.gov.au (V.D.); lachlan.webb@defence.gov.au (L.W.); andrew.rider@defence.gov.au (A.R.)

**Keywords:** supercapacitor, nanocomposite, energy materials, carbon nanotubes, multifunctional composites

## Abstract

Composite structural supercapacitors (SSC) are an attractive technology for aerospace vehicles; however, maintaining strength whilst adding energy storage to composite structures has been difficult. Here, SSCs were manufactured using aerospace-grade composite materials and CNT mat electrodes. A new design methodology was explored where the supercapacitor electrolyte was localised within the composite structure, achieving good electrochemical performance within the active region, whilst maintaining excellent mechanical performance elsewhere. The morphologies of these localised SSC designs were characterised with synchrotron X-ray fluorescence microscopy and synchrotron X-ray micro-computed tomography and could be directly correlated with both electrochemical and mechanical performance. One configuration used an ionogel with an ionic liquid (IL) electrolyte, which assisted localisation and achieved 2640 mW h kg^−1^ at 8.37 W kg^−1^ with a corresponding short beam shear (SBS) strength of 71.5 MPa in the active area. A separate configuration with only IL electrolyte achieved 758 mW h kg^−1^ at 7.87 W kg^−1^ with SBS strength of 106 MPa in the active area. Both configurations provide a combined energy and strength superior to results previously reported in the literature for composite SSCs.

## 1. Introduction

The need for creating material systems with greater volumetric and gravimetric efficiencies has led to increasing research effort in multifunctional materials and structures. Multifunctional materials reduce or eliminate the parasitic weight of functional components in traditional structures. One method to achieve multifunctional capability is for the functional components to become load-bearing entities within the structure [1,2,3]. Multifunctional composites have previously been explored in energy storage applications with batteries [4,5,6], dielectric capacitors [7,8] and supercapacitors [9,10,11,12]. Amongst these applications, supercapacitors, or electrical double-layer capacitors (EDLC), have gained attention due to the inherent construction simplicity and excellent cycle life [13]. Supercapacitors can also be fabricated with less hazardous materials when compared to batteries [14,15]. With the addition of reactive species at the electrode–electrolyte interface, supercapacitors can also generate additional pseudocapacitance, which can increase energy density [16,17]. Consequently, supercapacitors can possess high power density [18], along with high stability [19], and reversibility [20], which bridges the technology gap between batteries and dielectric capacitors. The potential for multifunctional composite structures to provide rapid energy transfer cycles over a wide temperature range, therefore making supercapacitors attractive for applications in the aerospace [4,21,22,23] and automotive industries [22,23], portable electronic devices [23], and civil construction [23].

Materials, such as carbon black and activated carbons, exhibit high specific surface area (SSA), in excess of 1500 m^2^ g^−1^ [24,25], which are desirable for electrodes used in EDLCs. However, high SSA powders are not easily adapted towards the fabrication of structural supercapacitors (SSCs), due to their poor mechanical performance and lack of long-range order [26]. Carbon nanotubes (CNTs) are an alternative electrode material, possessing high mechanical strength, excellent electrical conductivity, low density, and high electrochemical stability. An ideal form for CNTs for application in SSCs is achieved with mats, where long and intertwined CNTs have been applied as electrode, current collector, and mechanical reinforcement in multifunctional structures [27,28,29]. Despite these advantages, CNTs typically exhibit lower energy storage capacity due to an SSA less than 400 m^2^ g^−1^ [30,31,32,33,34]. CNT mats are also a suitable form for integration into carbon fibre-reinforced polymer (CFRP) composites for the production of SSCs [12,29] with tensile strength and Young’s modulus as high as 2090 MPa and 169 GPa, respectively [27,35].

Further consideration in the integration of supercapacitors into high performance CFRPs requires the use of materials that are compatible with the high temperature and pressure environments used in fabrication. Therefore, conventional aqueous and solvent-based electrolytes are unsuitable. However, ionic liquids (ILs) with inherent low vapor pressure, nonflammability, and high chemical and thermal stability are highly compatible with composites manufacturing. ILs also have favourable electrolyte properties, exhibiting a wide electrochemical window [36], and high ionic conductivity [37]. However, the liquid state of ILs may lead to problems in localisation [38,39], which may be alleviated when ILs are incorporated polymers [40]. Composite SSC researchers have sought to improve the mechanical performance of their supercapacitors by using various types of polymer electrolytes. CNT electrodes have been used with ionogel electrolytes comprised of polyvinylidene fluoride (PVDF)-based polymers and a range of different dopant ILs including imidazolium-based [32,39,41,42,43,44,45] and pyrrolidinium-based [12,46]. While this combination of CNTs and PVDF ionogels have been explored for the fabrication of SSCs previously, few electrolyte configurations have been explored for optimisation of both electrochemical and mechanical performance in composites aside from [12]. Previous SSC configurations have examined sandwiching a self-contained supercapacitor stack with ionogel electrolyte into a composite [12,47] or infusing the whole SSC and composite structure with an electrolyte/resin mixture as the matrix material [5,11,23,48,49,50,51,52,53,54,55,56,57]. Embedding a self-contained supercapacitor is structurally disruptive and produces localised increases in laminate thickness and stress concentrations. Similarly, the use of an electrolyte/resin matrix is limited by the mutually incompatible requirement of high ionic conductivity and mechanical strength, with the final component typically exhibiting relatively low mechanical strength and poor electrochemical performance. In addition, relatively little research has utilised aerospace-grade materials and autoclave processing needed for high performance composites [29,58]. While all the aforementioned studies on SSCs produced promising results, the mechanical interaction between the electrodes and the ionogel electrolyte is not well-characterised, and localisation of the discrete SSC elements within the composite has not been considered.

Our recent work demonstrated the fabrication of a composite SSC comprising of discrete CNT mat electrodes, a structural glass fibre separator, and an IL electrolyte incorporated into a CFRP composite [29,33]. The current paper develops this system further with the fully integrated supercapacitor incorporated into aerospace CFRP systems using different IL electrolyte localisation strategies, namely removal of resin from a discrete region of the glass fibre separator, and the use of ionogel electrolytes. To understand the internal chemistry and morphology of the different localisation configurations, synchrotron-based X-ray fluorescence microscopy (XFM) and X-ray micro computed tomography (XµCT) has been employed and correlated with trends in the electrochemical and mechanical performance. The research provides critical information in guiding the future design of high-performance multifunctional composite structures.

## 2. Materials and Methods

Electrodes consisted of interconnected predominantly multiwall carbon nanotubes and iron carbide nanoparticles, although there is evidence from our previous TEM studies that some single-walled CNTs are present in the as-received mats [29]. These CNTs are produced with a ferrocene-based floating catalyst chemical vapour deposition technique and flattened into CNT mats with a 10–15 g m^−2^ areal density and an average thickness between 20–30 μm (Huntsman, Houston, TX, USA). Hexforce 1080 glass fibre with 50 g m^−2^ areal density with Hexply 914 resin prepreg (1080/914) (Hexcel Corporation, New Haven, CT, USA) was used at the structural separator material. Poly(vinylidene fluoride-co-hexafluoropropylene) (PVDF-HFP) with an Mn of 110 kDa (Merck, Sydney, NSW, Australia), 98% 1-ethyl-3-methylimidazolium bis(trifluoromethylsulfonyl)imide ([EMIm][NTf_2_]) (Merck, Sydney, NSW, Australia), 98% 1-butyl-3-methylimidazolium tribromide ([BMIm][Br_3_]) (Chem-Supply, Adelaide, SA, Australia), 99.9% N,N-dimethylacetamide (DMAc) (Merck, Sydney, NSW, Australia), acetone, and methyl ethyl ketone (MEK) (Chem-Supply, Adelaide, SA, Australia) were directly used without further purification. IM7/977-3 prepreg tape (Solvay, Trenton, NJ, USA) was used to for supercapacitor structural reinforcement.

Ionogel films were prepared using a conventional solvent casting method. [BMIm][Br_3_] was utilised as the dopant IL for XFM studies, while [EMIm][NTf_2_] was employed for all other experiments. PVDF-HFP and ionic liquid (IL) at 70:30 *w*/*w* were dissolved and homogenised in DMAc at 25 wt% through magnetic stirring under ambient conditions for 5 h. The resultant mixture was then cast onto a flat Teflon-coated surface and the solvent evaporated at 80 °C for 24 h followed by drying at 80 °C in vacuo for 5 h to remove residual DMAc. The ionogel film thicknesses were adjusted to provide an equivalent areal density of IL, which ranged between 3 and 15 mg cm^−2^, with 12 mg cm^−2^ used for electrochemical and mechanical characterisation. The equivalent areal density was determined based on the total initial amount of IL (in mg) per a unit area of 5.07 cm^2^ (area of the 25.4 mm diameter ionogels).

Elemental mapping of the SSCs used the XFM beamline at the Australian Synchrotron, with the use of the microprobe end-station. Data was collected using a Maia 384-element planar silicon array detector, positioned in the backscatter geometry. SSCs were oriented flat and normal to the incident beam with an energy of 18.5 keV and spot sizes of 200 or 300 µm. The raster scan trajectory used a maximum horizontal velocity of 20 mm s^−1^. Data was analysed using the GeoPIXE software package [59] and post-processed with ImageJ (v1.53c) (National Institute of Health, Washington, MD, USA). The cured areal density of IL was determined using the quantitative concentration of IL (in mg) obtained from the XFM experiments, per a unit area of 5.07 cm^2^ in the centre region of the composite panels, for direct comparison with the initial areal density of IL.

XµCT experiments were performed at the Imaging and Medical Beamline (IMBL) at the Australian Synchrotron, with the use of a scintillator and pco.edge (PCO,DE) detector, using 52 keV monochromatic X-rays from a 3 T bending magnet. A total of 1800 images acquired over a 180° rotation were collected with a spatial resolution of 5.81 µm pixel^−1^. Samples prepared for imaging were 8 mm-wide strips cut through the full length of the SSC cores and embedded in 8 mm diameter cylindrical moulds with Araldite K3600 (Huntsman, US). Images were post-processed with Australian Synchrotron’s IMBL pre-processing software, and reconstructions were processed using Dragonfly software (v2021.1) (ORS, Montreal, QC, Canada) [60].

Electrochemical testing used a galvanostat/potentiostat VMP3 (BioLogic, Grenoble, ARA, France), with electrical connection to two 100 × 100 mm stainless steel plates used to clamp the SSCs. The active CNT mat electrode area was determined from digital images, which clearly showed the spread of the IL, to normalise electrochemical measurements. Cyclic voltammetry (CV) of the SSC cores was measured from 0 to 3 V at 10 mV s^−1^ and galvanostatic charge–discharge (GCD) cycling was measured from 0 to 3 V versus the counter electrode, with 2 to 20 mA charging and discharging currents. Electrochemical impedance spectroscopy (EIS) was measured from 1 MHz to 100 mHz with 10 mV RMS potential. Specific capacitance (*C_S_*), energy (*E_S_*), and power (*P_S_*) are key performance metrics to assess electrochemical performance and are calculated from GCD experiments according to the following formulae, respectively [16]:

(1) $$C_{S}=\frac{I\times t_{d}}{V_{d}\times m}\;$$

(2) $$E_{S}=\frac{C_{S}\times V_{d}{}^{2}}{2}\;$$

(3) $$P_{S}=\frac{E_{S}}{t_{d}}$$

where *I* is the discharge current, *t_d_* is the discharge time, *V_d_* is the discharge voltage drop not including the resistance drop of the GCD curve, and m is the combined mass of CNT mat cathode and anode activated by the IL electrode.

Interlaminar shear strength (ILSS) of the SSCs and baseline laminates was measured directly with a short beam shear (SBS) test according to ASTM D2344 [61]. The specimen length was cut to 6 t and the width was cut to 2 t, where t is sample thickness and b is the width. Composite SSC panels were manufactured to 1.5 mm thickness and the baseline 977-3 and Mid-1080/914-E to 3 mm, such that sample length and widths ranged between 9–18 mm and 3–6 mm, respectively. A minimum of four replicate samples were tested for the central SSC region with a minimum of eight tested for the baseline panels. Tests were conducted with an SBS test fixture, WTF-SB (Wyoming Test Fixtures Inc., Salt Lake City, UT, USA), at a loading span of 4 t ± 0.3 mm using a 6 mm diameter upper roller and two 3 mm lower rollers. Equation (4) was used to calculate the *SBS* strength *F^SBS^*:

(4) $$F^{SBS}=0.75\frac{P_{d}}{b\times t}$$

where *P_d_* was the load at which the first load drop occurred, indicating interlaminar failure at the weakest interface.

## 3. Results and Discussion

### 3.1. Manufacture of Supercapacitor Panels

**Figure 1:**
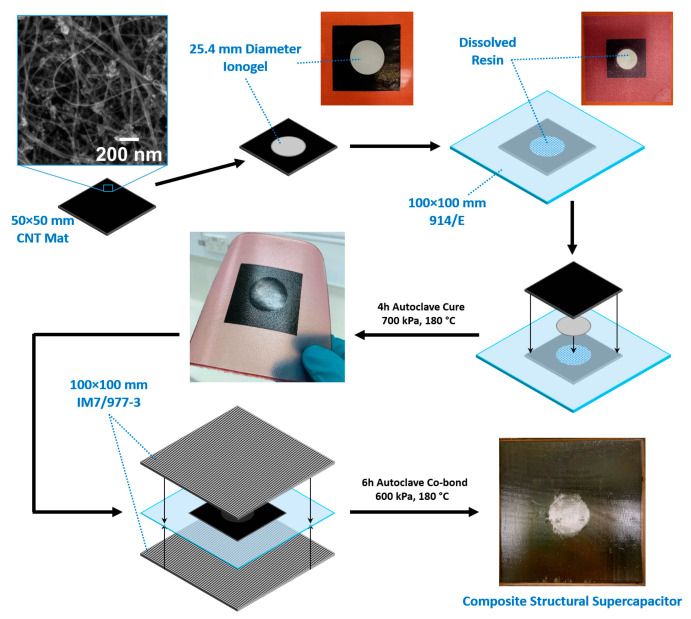
Typical fabrication sequence for structural supercapacitor (SSC), showing photos of localised SSC core layup with CNT mat electrode, with inset electron micrograph, and ionogel, glass fibre separator, and the final cured core and composite SSC with 25 mm electro-activated region in the centre.

Five different structural composite device configurations were manufactured to examine the relative electrochemical and mechanical performance of the SSC using a CNT mat as the electrodes and a 1080/914 glass fibre separator. The description of each configuration is shown in Table 1. Localised composite SSCs can be scaled to any size; in this work, the materials were designed for a 25.4 mm diameter electro-active region of SSC. In configurations SSC2 to SSC5, a 25.4 mm diameter disk of resin was removed to improve ionic conductivity and allow interpenetration of the ionogel films with the glass fibre separator layer during cure. The schematic for a typical manufacturing process of these SSCs is shown in Figure 1, designed to create a 25.4 mm diameter electro-active region. The electrode must be oversized in relation to the designed electro-active region to ensure maximum capacitance and load transfer through the CNT mat electrode, in which a 50 × 50 mm dimension was selected for all supercapacitor configurations.

The core of SSCs panels was prepared by autoclave curing the CNT mat and 1080/914 fibreglass separator with or without ionogels, using a silicone rubber sheet as a caul plate, at 180 °C and 700 kPa pressure for 4 h. In some configurations, the 1080/914 glass fibre separator had a dissolved resin section located centrally for placement of the ionogel films. Electrochemical testing was typically completed for the core SSC panels prior to co-bonding with 6 plies of IM7/977-3 plies either side at 180 °C and 600 kPa pressure for 8 h for mechanical characterisation. XFM samples used 1 ply of IM7/977-3 that was added either side of the SSC1 to SSC3 configurations and cured simultaneously to simulate the processes that occur in one-shot manufacture. A series of baseline panels were also produced with 24 plies of IM7/977-3 without ionogel or IL. The series comprised IM7/977-3 only, with a midplane of 1080/914, with CNT mat plus 1080/914 without (Dry SSC1) and with (Dry SSC2) the central resin disk removed from the 1080/914 layer.

### 3.2. Ionic Liquid Localisation

**Figure 2:**
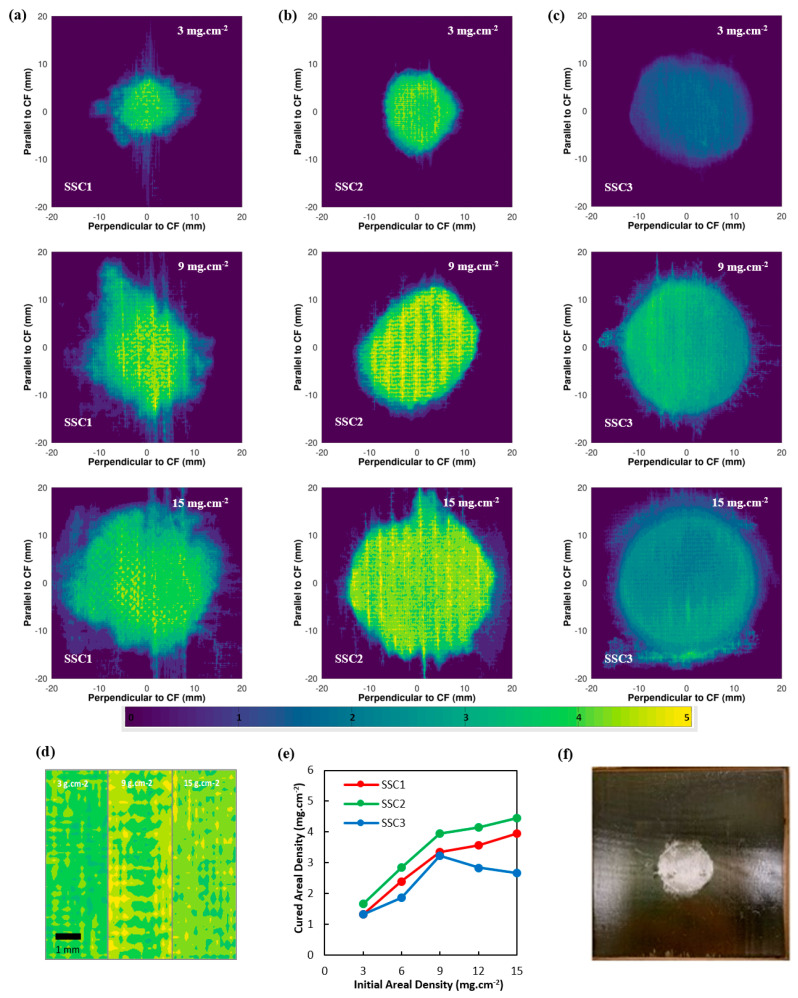
Elemental maps collected by synchrotron XFM showing the distribution of Br in the structural supercapacitor for (**a**) SSC1, (**b**) SSC2, and (**c**) SSC3 configurations. [BMIm][Br_3_] was introduced directly (SSC1 and SSC2) or within circular ionogel films of 25.4 mm diameter, with an initial areal density ranging from 3 to 15 mg cm^−2^, (**d**) high magnification maps for SSC2. Colour scale represents the per-pixel cured areal density of Br in mg cm^−2^, (**e**) the final normalised areal density of Br for SSC1, SSC2, and SSC3 configurations plotted as a function of the initial areal density of [BMIm][Br_3_]. (**f**) Fabricated SSC3 with IM7/977-3 encapsulation revealing the central IL rich region.

The three IL localisation strategies shown in Figure 2 reveal both clear and subtle differences, which are possible to observe due to the XFM data providing through-thickness, chemical concentration information at high spatial resolution. Figure 2a shows SSC1, where IL is added directly to the glass fibre prepreg and CNT electrodes, contrasting with Figure 2b which shows SSC2, where IL is added to the glass fibre prepreg with a 25.4 mm disk of resin removed. In Figure 2c, the IL was introduced through the 25.4 mm diameter ionogels placed into the glass fibre prepreg with the resin removed. These ionogels help to localise the IL, creating a discrete electro-active supercapacitor region by providing a porous volume for the IL to occupy, which is unlikely to be backfilled by the resin system during the cure cycle. These concentrations of electrolyte improve ionic conductivity of the supercapacitor as seen later in the electrochemical testing.

In SSC1, the IL distribution evolves in a more heterogeneous pattern compared to SSC2, which is directly related to the reduced free volume for IL diffusion in SSC1. Both SSC1 and SSC2 reveal the IL also concentrates in a fine pattern that replicates the imprint for the plain-weave glass fabric, suggesting some IL migration in the warp and weft directions. Notably, the IL is seen to have preferentially diffused into the outer carbon fibre prepreg engineered vacuum channels (EVaCs) during the vacuum debulk stage and then intermixed with the surrounding resin during cure. This flow of the IL away from the central electro-active region is detrimental to the surrounding epoxy matric mechanical performance and deprives the SSC of some IL, which could have improved the IL concentration in the centre of the device. The IL diffusion into the EVaCs is more pronounced for the SSC2 compared to the SSC1 configuration and reaches a maximum for the 9 mg cm^−2^ starting concentration. Figure 2d shows higher resolution progression of the IL diffusion as a function of concentration for SSC2, with the IL building up in the EvaCs from 3 to 9 g cm^−2^ and then a migration assisted by the cross-ply fibres in the glass prepreg leading to a more even distribution at 15 g cm^−2^. A similar trend is observed in SSC1, however, the presence of resin in the glass prepreg layer leads to a more heterogeneous dispersion of the IL in a lateral, rather than through-thickness, direction. Consequently, at 15 g cm^−2^ the SSC1 average IL concentration in the central region is lower than SSC2, as shown by the plot in Figure 2e. In SSC1, there is also a greater spread of IL at lower concentrations beyond the central region that is facilitated by greater penetration into the EVaCs, but also indicated by the evolution of lateral fissures, potentially caused by higher localised pressure. The XFM data suggests that IL movement during fabrication of the SSCs follows an initial stage of through-thickness diffusion to fill the free-space and EVaC channels during vacuum debulking, followed by resin from the carbon and glass prepreg layers backfilling any free volume, not occupied by the IL, when the resin flows during cure. The removal of the resin disk in the glass prepreg appears to facilitate localised through-thickness diffusion of the IL, leading to a more a uniform, higher concentration in the central region that simultaneously reduces the level of resin back-filling, enabling higher specific power.

In Figure 2c, SSC3 reveals the lowest IL concentration and relatively small differences in the 9 and 15 g cm^−2^ concentrations, also confirmed by data in Figure 2e. The requirement to increase the ionogel thickness to achieve a higher equivalent areal concentration has the effect of compressing the polymer under vacuum debulking and reducing the volume available for the IL within the film, which will significantly impact the ionic conductivity and specific power of the supercapacitor. Consequently, some of the IL diffuses to the perimeter of the ionogel disks where the gap with the resin border exists, creating a uniform ring with higher concentration of IL than either SSC1 or SSC2 configurations at this location.

**Figure 3:**
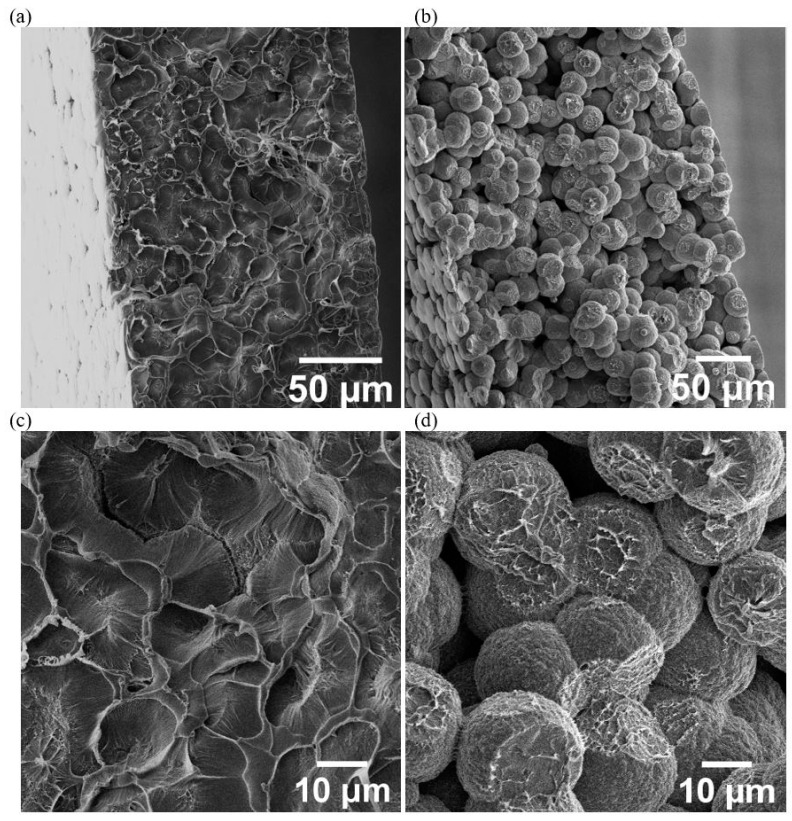
SEM micrographs of PVDF-HFP ionogel in cross-section after autoclave cure cycle where, (**a**,**c**) is the as-cast ionogel and (**b**,**d**) 50 µL of additional IL was added to the ionogel before cure.

**Figure 4:**
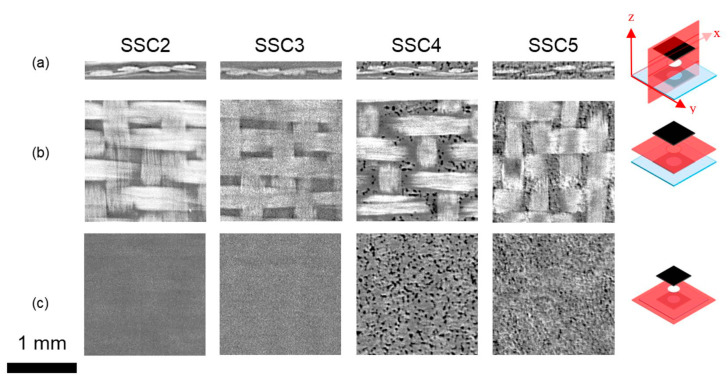
XμCT cross-sections of SSC2 to SSC5. (**a**) Cross-section taken through the x-z plane, while (**b**,**c**) show cross-sections taken through the x-y planes at the centre of the glass layer and at the electrolyte/CNT electrode interface, respectively. The x-y-z orientation and section location are indicated at the right-hand side.

In terms of anticipating the best electrochemical performance from the IL distribution observed in Figure 2, it might be expected that SSC2 would be superior, as the localised and higher IL concentration should provide greater ionic conductivity. In SSC3, the ionogel would not be expected to perform as well electrochemically; however, the film does appear to provide a more uniform lateral diffusion and, potentially, the porous PVDF polymer might provide a greater barrier to IL and resin mixing during cure. The ionogel configuration would require a method to increase the IL concentration in a thinner film. To explore this possibility further, an ionogel film with 12 mg cm^−2^ of IL had an additional 50 mL of IL added prior to the autoclave cure. Figure 3 shows cross-sections for cured ionogels with and without the additional IL. In Figure 3b,d, a porous structure has been achieved the PVDF-HFP, with the spherical particles providing clear space for IL diffusion. In contrast, Figure 4 show the ionogel after autoclave cure has become compressed, with adjacent particles coalescing and providing limited space for IL movement. Consequently, the additional 50 mL of IL added to the ionogel became the configuration tested in SSC4 and SSC5, detailed in Table 1.

### 3.3. Synchrotron X-ray Micro Computed Tomography (XµCT)

Figure 4 presents XμCT data for SSC2 to SSC5 configurations, with 2D cross-sections taken from the 3D reconstructed tomogram used to interrogate the location and porosity of the 25.4 mm diameter ionogel disks within SSC3 to SSC5, in comparison to SSC2 without the ionogel, as detailed in Table 1. SSC2 and SSC3 show similar cross-sections, suggesting the porosity within the ionogel is not resolvable or absent.

The SEM images shown in Figure 3a,c would suggest that the morphology in SSC3 would be difficult to detect based on the morphology of the polymer film produced after cure. Whilst Matsumoto [62] and Shirshova [63] could resolve the bi-continuous phase morphology, the porosity observed in the SSC4 and SSC5 ionogels, as shown in Figure 4, needed to be enhanced by solvent extraction of the IL prior to imaging. In Figure 4, SSC4 and SSC5 both exhibit clear through-thickness porosity with SSC4 showing fewer and larger individual pores ranging from 10 to 30 μm diameter, which will improve the ionic conductivity. However, a more interconnected network of smaller pores is more desirable, as this improves the shear strength of the ionogel. This is consistent with the porosity observed in the isolated ionogel imaged in Figure 3b,c. There is also evidence of larger pores more than 60 μm diameter forming through coalescence of the smaller pores. The additional 50 μL of IL added to the electrodes and separator in SSC5 reveals the evolution of a higher density and finer porosity; however, as the pores appear to be close to the resolution limit of the reconstructed tomogram, approximately 5.81 µm pixel^−1^, this cannot be stated with confidence.

**Figure 5:**
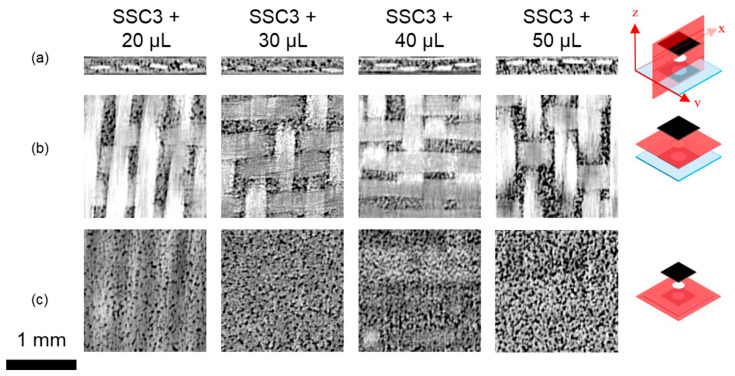
XμCT cross-sections of SSC3, showing the effect of increased EMIm TFSI IL loading on the porous ionogel microstructure with (**a**) cross-section taken through the x-z plane, while (**b**,**c**) show cross-sections taken through the x-y planes at the centre of the glass layer and at the glass layer/CNT electrode interface, respectively. The x-y-z orientation and section location are indicated at the right-hand side.

To confirm the trends observed in Figure 3 and Figure 4—which suggest additional IL added to the cast ionogel enables the PVDF-HFP porous structure to be developed and increases the quantity of IL and, by implication, the ionic mobility in the SSC—a series of SSC3 configurations were examined with additional amounts of IL added to the cast ionogels prior to cure. Figure 5 shows a clear trend of increasing ionogel porosity as a function of additional IL concentration. The size of the individual pores does not appear to change significantly for the different ionogels, but as the population increases, the merged pores create larger apparent pore structure, which should increase the ionic conductivity and power. It should be noted that as the PVDF-HFP melts during cure and thus forms a biphase system with the IL, the porosity observed in Figure 3, Figure 4 and Figure 5 represents the phase separated structure that forms with the IL during solidification of the polymer after curing. Consequently, it is expected the volume of the IL relative to the solid phase, and hence the apparent pore size, would increase with larger addition of IL.

### 3.4. Electrochemical Performance

**Figure 6:**
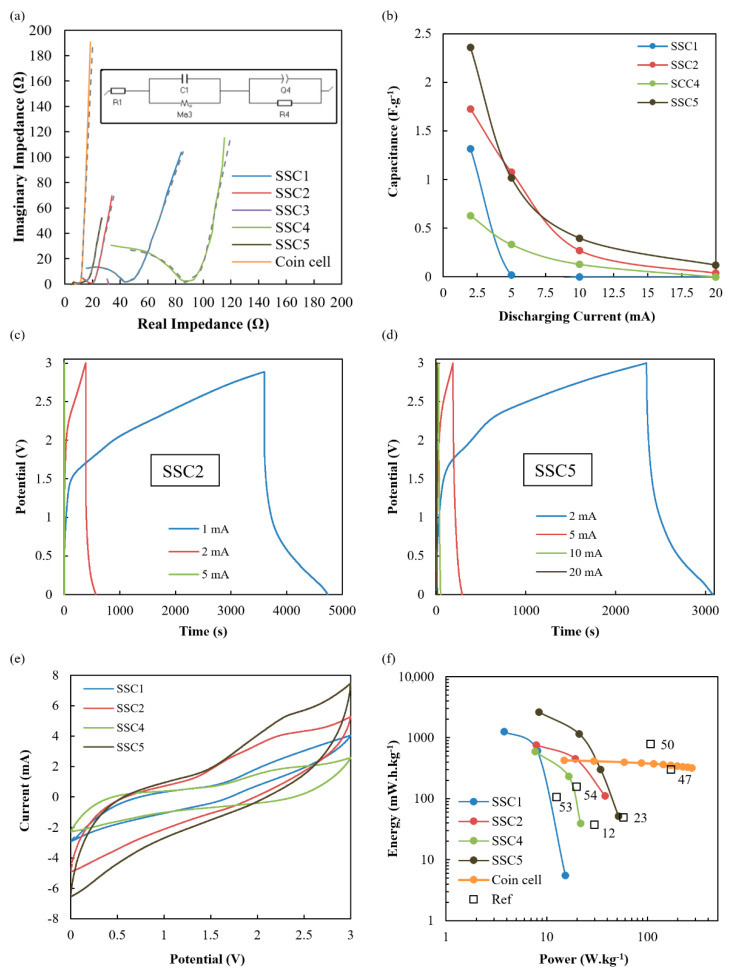
Electrochemical performance of SSC cores normalised to the device mass of the activated SSC core including electrode, separator, ionogel and electrolyte; (**a**) EIS from 100 mHz to 1 MHz with inset equivalent circuit and grey dashed lines for the equivalent circuit fits, (**b**) GCD capacitance vs. discharge rate, increasing current GCD of (**c**) SSC2 and (**d**) SSC5, (**e**) cyclic voltammogram at 10 mV s^−1^, and (**f**) Ragone plot including SSC and 2023 coin cell results in comparison with high-performance composite SSCs in literature [12,23,47,50,53,54].

The results from the electrochemical testing of SSC1 to SSC5 configurations detailed in Table 1 are shown in Figure 6. The data reveals a consistent trend with SSC2 and SSC5 exhibiting the best performance, whilst SSC1 and SSC4 are functional but quite inferior, whereas SSC3 did not have enough conductivity to effectively cycle, so the results could not be included. The explanation for this trend is consistent with the observed morphology of the ionogel films detailed in Figure 3, Figure 4 and Figure 5 and the XFM data in Figure 2. The electrochemical impedance spectroscopy (EIS), as shown in Figure 6a, has been analysed with equivalent circuit modelling, where the fitting data is plotted as dashed grey lines and the equivalent circuit used for all datasets is shown inset in Figure 6a. Table 2 shows the equivalent circuit component values for the plots in Figure 6a. The equivalent circuit used to accurately model all cells incorporates electrical components used as analogues to physical characteristics of the electrochemical cell. Of particular note are the three resistive components: the equivalent series resistance (R1), the charge transfer resistance (R4), and the diffuse layer resistance (Rd3). Rd3 is extracted from the restricted diffusion element (Ma3) used to model the restricted ion movement through the diffuse region near the electrode, particularly relevant in these SSCs where epoxy and ionogel polymers restrict the accessibility of ions to active electrode sites. From the Nyquist plot, Figure 6a, and Table 2, we see that SSC5 showed the lowest charge transfer resistance due to the greatest ionogel porosity, as seen in Figure 4, and total volume of IL electrolyte. Rd3 was generally higher for SSC4 and SSC5 due to the restriction of the ionogel at the electrode, but decreased with additional IL electrolyte addition. The baseline coin cell with the same electrode and separator shows higher R1 due to the coin cell internal connections, higher C1 and C4 and lower R4 due to the increased IL concentration, and a much less restricted electrolyte region between the electrodes.

The higher and more localised IL concentration in SSC5 and SSC2, as determined by XFM, shown in Figure 2b, and XμCT, shown in Figure 4, also leads to higher capacitance as a function of discharge current (Figure 6b), improved current–voltage window (Figure 6e), and improved energy and power density (Figure 6f). GCD with increasing current of SSC2 shows significant IR drop and large capacitance with the long-sloped discharge curve (Figure 6c), and SSC5 similarly shows large capacitance with pseudocapacitive plateau at 1.8 V on the charge curve and 0.5 V on the discharge curve. There are also pseudocapacitive peaks identified in Figure 6e, which make a significant contribution to the capacitance that is associated with fast redox reactions involving the CNTs and iron nanoparticles distributed throughout the CNT mat electrode [29]. The cycling stability of SSC2 and SSC5 are shown in Figure S1, indicating a significant loss of capacitance after only 100 cycles, due to moisture ingress over the course of days into the composite, causing hydrolysis of the water above 1.5 V during cycling. Future testing of these composite SSCs will incorporate additional composite plies on both sides of the embedded device and a thin thermoplastic film with ultralow moisture and oxygen permeability to eliminate this effect. The cyclic voltammograms of SSC2 and SSC5 are shown in Figure S2a,b, respectively, with increasing cycling rate from 10 to 100 mV s^−1^ where the capacitive contribution of this pseudocapacitive process is seen to diminish at higher cycling rates due to the limited reaction kinetics. Both figures show a resistive slope associated with the limited ionic mobility of the SSC morphology; however, SSC5 shows higher capacitance even at high cycling rates.

The additional benefit of the increased and more localised IL concentration in SSC5 and SSC2 is reflected in the energy and power density in Figure 6f, with SSC5 producing 2640 mW h kg^−1^ at 8.37 W kg^−1^ and SSC2 producing 758 mW h kg^−1^ at 7.87 W kg^−1^. Whilst both configurations have relatively similar quantities of IL added prior to cure, the improved performance of the ionogel may be related to the configuration reducing mixing between the IL and epoxy during cure and, consequently, limiting the reduction in IL ionic mobility this mixing causes. The corresponding deterioration in performance for SSC1 and SSC4, as shown in Figure 6f, also correlates with the reduction in localised IL concentration shown in Figure 2 when the resin is left in the glass fibre prepreg for SSC1 and the reduced porosity of the ionogel shown in Figure 5 for SSC4. Similarly, in SSC3 where XFM showed the lowest IL concentration and SEM cross-sections revealed a low-porosity structure, shown in Figure 3a,c, the poor performance would be predicted.

The performance of these SSC configurations is compared with a 2023 coin cell made from CNT mat and glass separator and the same IL in Figure 6f. The coin cell has a lower specific energy but can achieve much higher specific power, due to a significantly lower equivalent series resistance, shown in Figure 6a, as there is no epoxy polymer or ionogel restricting ion movement. In SSC5, the relatively thick ionogel film lowers ionic conductivity, and in SSC2 the intermixing with the epoxy during cure is likely to affect the ionic mobility. However, the encouraging aspect of the SSC5 configuration is the high energy density compared to composite SSC literature with very good specific power performance, as shown in Figure 6f. The high specific energy for SSC5, relative to the coin cell, also suggests that the high-pressure consolidation is beneficial for electrochemical performance. Future work developing localised composite SSCs will examine the reduction in ionogel thickness to decrease the ionic resistance and improve the power output at higher discharge rates.

### 3.5. SSC Mechanical Performance

**Figure 7:**
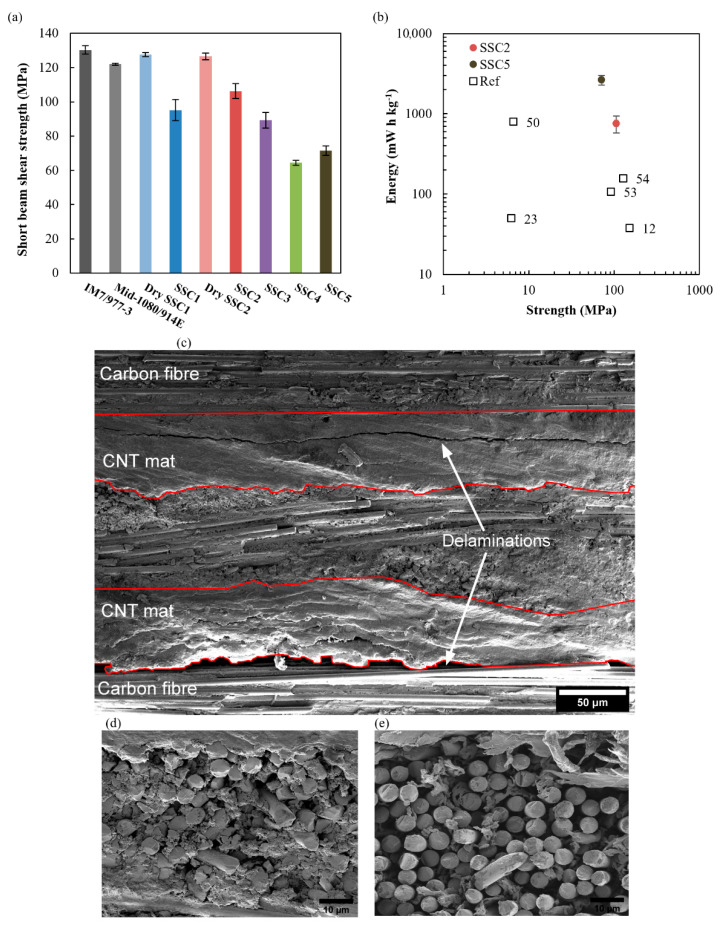
Mechanical performance of the baseline and SSC configurations showing (**a**) the SBS strength, (**b**) strength vs. energy plot with literature comparison [12,23,50,53,54], (**c**) delamination failure of SSC2, and (**d**,**e**) the morphology of the epoxy resin and ionogel matrices within the fibreglass separator layer for SSC2 and SSC5, respectively.

Figure 7a shows the results of the SBS testing for the co-bonded SSC and baseline panels. The strength was calculated at the point at which the first load drop was observed, corresponding to interlaminar shear failure at the weakest interface. The stress field under continued loading is affected by the presence of damage, such that the peak load cannot be used to calculate an ILSS in a laminate with the soft inclusions of the SSC. All the baseline panels failed catastrophically without load drops prior to peak load. The laminate incorporating the layer of fibreglass at its mid-plane exhibited the lowest strength, with the strength of the other baseline panels in accordance with the 128 MPa manufacturer specification for the prepreg material [64]. The 6% lower strength of the configuration with fibreglass in the midplane (Mid-1080/914E) is potentially a consequence of resin-rich regions at the carbon-glass interface. Interestingly, the strength is restored by the addition of CNT mat between the carbon and fibreglass plies in Dry SSC2, which may act to increase the interfacial toughness [65]. Unexpectedly, removing resin from the fibreglass in the Dry SSC2 configuration did not reduce the SBS strength.

The SBS of the two functional laminates without ionogel, SSC1 and SSC2, decreased by 27% and 19%, respectively, which is attributed to IL mixing with the epoxy resin and forming a bi-phase microstructure [63]. Porous epoxy is visible between the glass fibres of a failed SSC2 sample in Figure 7d, which cannot be resolved by the XµCT shown in Figure 4. The knockdown is less severe for SSC2 because the prior removal of epoxy localises this process by creating open volume between the fibreglass tows; this allows a greater proportion of the baseline epoxy ILSS to be maintained because less surrounding resin matrix is compromised by IL during consolidation under pressure. Interlaminar shear failure initiates at either the interface between the primarily load bearing carbon fibre plies and the CNT mat or along a planar discontinuity within the CNT mat that exists due to the layered structure created by the manufacturing process, as shown for SSC2 in Figure 7c. It is worth noting that this failure morphology was broadly consistent across the functional samples because the softer ionogel separator layer does not carry a significant portion of the shear load in bending.

Although the addition of ionogel in SSC3 again improves the localisation of the IL, its strength is similar to SSC1 with a 31% knockdown with respect to the carbon baseline. PVDF-HPF is chemically incompatible with epoxy such that the SBS strength within the ionogel region is primarily determined by mechanical interlocking of CNTs bridging the interface between the epoxy resin and gel matrices; the morphology of this gel matrix is shown in Figure 7e for a failed SSC5 sample. The ILSS of SSC4 and SSC5 is further reduced to approximately half that of the baseline, with the detrimental effects observed in SSC1 and SSC3 combined because the excess free IL is not fully encapsulated in the ionogel. Additionally, the IL, which is localised in the ionogel, is still shown to remain mobile within the interconnected porous network of SSC5, Supplementary Video S1, enabling the high ionic conductivity in this configuration. Figure 7b highlights the improvements in energy density and shear strength achieved with SSC2 and SSC5 configurations, while localising the IL electrolyte within the SSC compared with results from the literature. It is worth noting that this work uses SBS strength as a measure of the structural performance of SSCs, differing from other work in the literature, which typically report the in-plane tensile, compressive, or shear strength and moduli [12,23,50,53,54,55]. ILs are known to compromise the mechanical integrity of epoxy resin systems [66], with the most significantly deleterious effect evident in fibre-matrix interfacial strength and the interlaminar shear strength for which the short beam shear test provides a direct measure. ASTM D3039 and D3518 do not directly interrogate these critical interfaces, with the reported strength and stiffness knockdowns predominately attributable to the use of softer materials and the increased cross-sectional areas associated with the added thickness of functional regions that do not contribute significantly to the load carrying capacity of the laminates.

## 4. Conclusions

The precise characterisation of the chemical and morphological condition of SSCs, made possible by high-fidelity synchrotron-based XFM and XμCT, has enabled a direct correlation to be made with both electrochemical and mechanical performance. Further, the characterisation has guided changes in the manufacturing processes and led to significant performance improvements for SSCs. Experimentation has confirmed that ionogels that have been post-doped with additional ionic liquid (IL) can then form a porous separator layer after high pressure and temperature processing. Uniquely, the creation of the porous ionogel structure is facilitated by both the high IL concentration and processing conditions. The ionogel’s PVDF-based polymer prevents resin mixing, and the high-pressure consolidation maximises the interaction between the IL and CNT mat electrodes, leading to a high energy density. The ionogel provides good electrochemical performance but leads to some reduction in mechanical strength due to the absence of the IL/epoxy bi-phase structure within the separator layer that forms if resin mixing occurs. However, an alternative configuration, without ionogel, where IL is placed directly in the separator layer region, where the resin has been removed, provides a compromise to the ionogel. This configuration cannot prevent IL and epoxy mixing, leading to the formation of a biphase epoxy/IL structure. The biphase structure improves mechanical strength, but also leads to some reduction in electrochemical performance due to the IL ionic conductivity being compromised by the epoxy resin. Nevertheless, both configurations show very good mechanical and electrochemical performance, relative to SSCs reported in the literature. The current work confirms that the approach of discrete placement of localised supercapacitor elements within a high-performance composite can be achieved with materials and processes that are compatible with, and favoured by, the high temperature and pressure environments required for manufacturing.

## Figures and Tables

**Table 1:** Structural supercapacitor configurations examined.

Designation	1080/914 Separator	Ionogel (12 mg cm^−2^)	Additional IL (50 µL)
SSC1	unmodified	-	Electrodes + separator
SSC2	central 25.4 mm diam. resin disk removed	-	Electrodes + separator
SSC3		2 × 25.4 mm disks either side of separator	-
SSC4			Ionogels
SSC5			Electrodes + Separator + ionogels

**Table 2:** Equivalent circuit component values.

Component	Physical Interpretation	SSC1	SSC2	SSC4	SSC5	Coin Cell
R1 (Ω)	Equivalent series resistance	~0.10	~0.10	~0.10	~0.10	8.2
C1 (uF)	EIS equilibrium EDLC	32	44	18	260	4800
Rd3 (Ω)	Diffuse layer resistance	25	8.5	34	13	20
t3 (s)	RC time constant	0.25	0.14	0.40	0.33	0.060
a3	Limited diffusion exponent	0.80	0.87	0.88	0.89	0.94
C4 (nF)	EIS equilibrium pseudocapacitance	9.7	3.9	3.1	29	19
R4 (Ω)	Charge transfer resistance	44	19	86	14	2.7

## Data Availability

The data presented in this study are available in the Supplementary Materials.

## Supplementary Materials

The following supporting information can be downloaded at: https://www.mdpi.com/article/10.3390/nano12152558/s1, Supplementary Materials: Figure S1. Galvanostatic charge-discharge cycling stability curves of SSC in air at 1 mA charge and discharge for (a) SSC2 and (b) SSC5. Figure S2. Cyclic voltammograms of SSC cores for (a) SSC2 and (b) SSC5.; Video S1: SBS testing of SSC5.
