# Supplementary material for: Composite Structural Supercapacitors: High-Performance Carbon Nanotube Supercapacitors through Ionic Liquid Localisation
Source: Nanomaterials (Basel). 2022 Jul 25;12(15):2558. doi: 10.3390/nano12152558 (PMC9330893; doi:10.3390/nano12152558)
Supplement: Supplementary file 1 [file nanomaterials-12-02558-s001.zip › nanomaterials-1814123 - SM Proofreading.pdf]

Supplementary Material

Composite Structural Supercapacitors: High-Performance Carbon Nanotube Supercapacitors through Ionic Liquid Localisation

Benjamin Mapleback*, Vu Dao, Lachlan Webb and Andrew Rider

Defence Science and Technology Group, Platforms Division, 506 Lorimer St., Melbourne, VIC 3207, Australia; vu.dao@defence.gov.au (V.D.); lachlan.webb@defence.gov.au (L.W.); andrew.rider@defence.gov.au (A.R.)
* Correspondence: benjamin.mapleback1@defence.gov.au; Tel.: +61-3-9344-2013

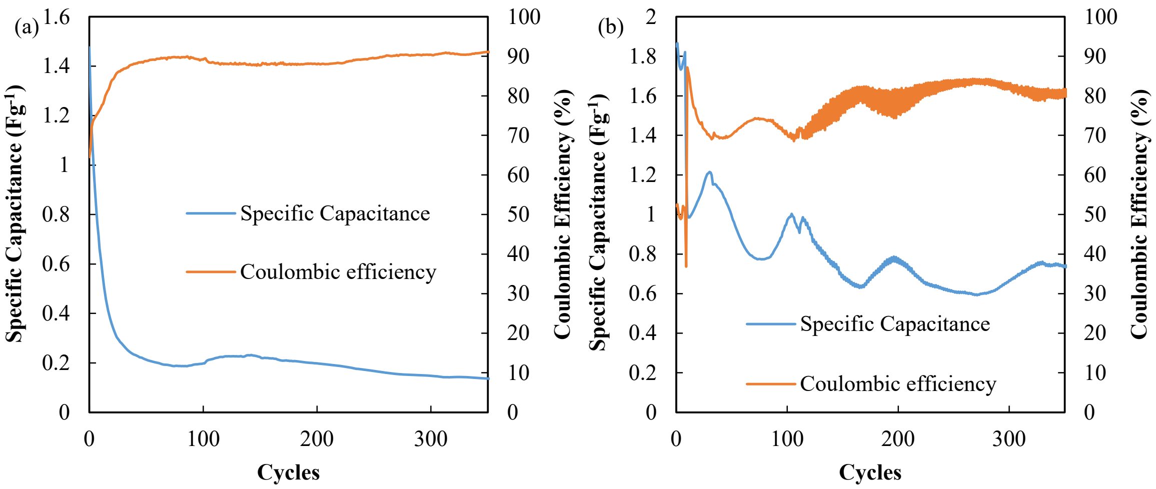

Figure S1. Galvanostatic charge-discharge cycling stability curves of SSC in air at 1 mA charge and discharge for (**a**) SSC2 and (**b**) SSC5.

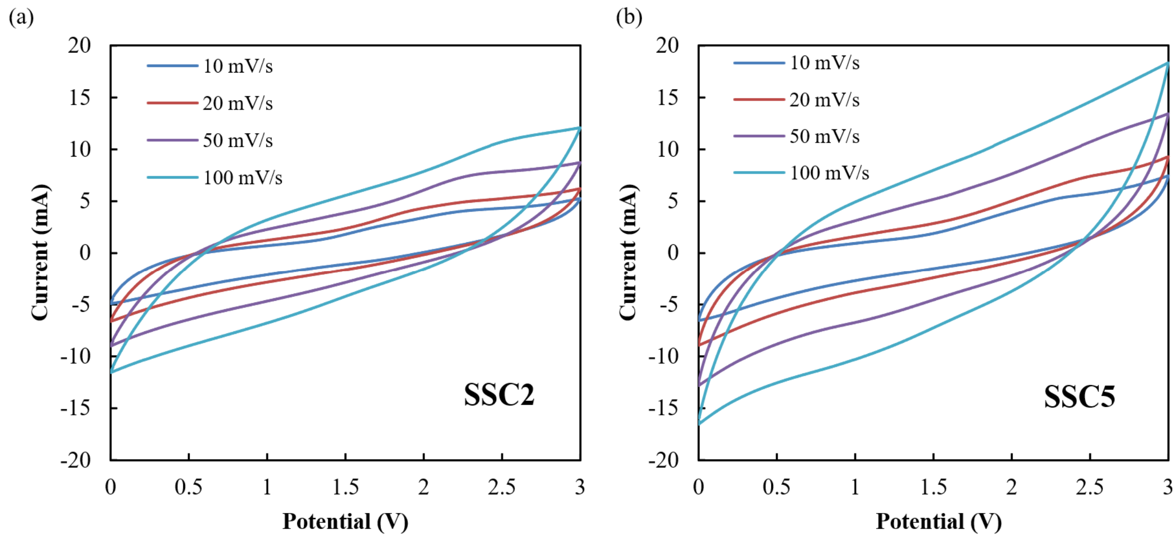

Figure S2. Cyclic voltammograms of SSC cores for (**a**) SSC2 and (**b**) SSC5.
